# Etiology and Pathology of Dysentery*From a paper read before the American Association of Physicians, Washington, D. C., May, 1892.—*The American Thelapist*.

**Published:** 1892-09

**Authors:** W. T. Councilman


ETIOLOGY AND PATHOLOGY OF DYSENTERY.*—We can divide dysentery into three forms: 1. Diphtheritic; 2. Catarrhal; 3 Amœbic.

Diphtheritic dysentery is characterized by necrosis of the epithelium and a fibrinous exudation. It is the form usually met with in acute epidemics. It may also appear in the course of a number of diseases, and may be produced by a number of causes. There is nothing in the anatomical lesions by which we may distinguish the action of a definite pathogenetic agent.

Catarrhal dysentery is characterized by an inflammation of the mucous surface of the intestine, leading to the produc-

*From a paper read before the American Association of Physicians, Washington, D. C., May, 1892.—*The American Thelapist.*

tion of shallow ulcers. Affections of the lymph follicles are more common in the catarrhal than in the other forms. What is said of the croupous is also the case in the catarrhal. In both these forms abscess of the liver may appear, but it is very rare.

Amœbic dysentery is characterized by definite lesions in the large intestine and elsewhere. The lesions have always the same character, and we can recognize in them the action of a common agent There are extensive ulcerations of the intestines, which, contrary to those of the other two forms, appear to be produced, not by extension downwards from the surface, but by a primary infiltration of the submucosa, with subsequent destruction of the overlying mucous membrane. Clinically, the disease is characterized by remissions and great chronicity. Abscess of the liver and lung is more frequent in amœbic dysentery. The disease is caused by the amœba dysentericæ, and is associated with most of the cases of tropical dysentery.—W. T. COUNCILMAN, M. D.

